# The ability to induce heat shock transcription factor-regulated genes in response to lethal heat stress is associated with thermotolerance in tomato cultivars

**DOI:** 10.3389/fpls.2023.1269964

**Published:** 2023-10-05

**Authors:** Junya Mizoi, Daisuke Todaka, Tomohiro Imatomi, Satoshi Kidokoro, Tetsuya Sakurai, Ken-Suke Kodaira, Hidehito Takayama, Kazuo Shinozaki, Kazuko Yamaguchi-Shinozaki

**Affiliations:** ^1^ Department of Applied Biological Chemistry, Graduate School of Agricultural and Life Sciences, The University of Tokyo, Tokyo, Japan; ^2^ RIKEN Center for Sustainable Resource Science, Yokohama, Japan; ^3^ Interdisciplinary Science Unit, Multidisciplinary Science Cluster, Research and Education Faculty, Kochi University, Nankoku, Japan; ^4^ The KAITEKI Institute, Inc., Tokyo, Japan; ^5^ Gene Discovery Research Group, RIKEN Center for Sustainable Resource Science, Tsukuba, Japan; ^6^ Institute for Advanced Research, Nagoya University, Nagoya, Japan; ^7^ Research Institute for Agricultural and Life Sciences, Tokyo University of Agriculture, Tokyo, Japan

**Keywords:** heat stress, *Solanum lycopersicum*, thermotolerance, cultivars, expression biomarker, heat shock response, transcriptome

## Abstract

Heat stress is a severe challenge for plant production, and the use of thermotolerant cultivars is critical to ensure stable production in high-temperature-prone environments. However, the selection of thermotolerant cultivars is difficult due to the complex nature of heat stress and the time and space needed for evaluation. In this study, we characterized genome-wide differences in gene expression between thermotolerant and thermosensitive tomato cultivars and examined the possibility of selecting gene expression markers to estimate thermotolerance among different tomato cultivars. We selected one thermotolerant and one thermosensitive cultivar based on physiological evaluations and compared heat-responsive gene expression in these cultivars under stepwise heat stress and acute heat shock conditions. Transcriptomic analyses reveled that two heat-inducible gene expression pathways, controlled by the heat shock element (HSE) and the evening element (EE), respectively, presented different responses depending on heat stress conditions. HSE-regulated gene expression was induced under both conditions, while EE-regulated gene expression was only induced under gradual heat stress conditions in both cultivars. Furthermore, HSE-regulated genes showed higher expression in the thermotolerant cultivar than the sensitive cultivar under acute heat shock conditions. Then, candidate expression biomarker genes were selected based on the transcriptome data, and the usefulness of these candidate genes was validated in five cultivars. This study shows that the thermotolerance of tomato is correlated with its ability to maintain the heat shock response (HSR) under acute severe heat shock conditions. Furthermore, it raises the possibility that the robustness of the HSR under severe heat stress can be used as an indicator to evaluate the thermotolerance of crop cultivars.

## Introduction

Due to global warming, the frequency and severity of heat waves are increasing (FAO, 2016). Accordingly, there is a growing demand for thermotolerant cultivars of various crop species. Tomato is one of the most important fruit crops in the world. Although tomato is a relatively heat-resistant crop, extreme heat stress conditions negatively affect its production in the field. In the case of high value-added tomatoes, such as those for fresh eating, the growth environments are controlled, but even then, much energy is needed for cooling. Therefore, it is important to select or develop thermotolerant cultivars for sustainable tomato production.

Tomatoes are subjected to various types of physiological damage at each stage of growth due to high-temperature stress (Hoshikawa et al., 2021). During the seedling and vegetative growth stages, the photosynthetic system is known to be damaged by high-temperature stress. In particular, photosystem II is known to have its maximum quantum yield (Fv/Fm) reduced, and the Fv/Fm value correlates with high-temperature tolerance (Zhou et al., 2015). Heat-induced damage to leaves leads to cell death, which negatively affects growth. Furthermore, during reproductive growth, high-temperature stress causes the abortion of flowers. This is due to the sensitivity of pollen development and maturation processes to high-temperature stress, and the viability of pollen has also been shown to be related to thermotolerance (Bita et al., 2011; Zhou et al., 2015). This damage at the organ level is fundamentally due to cellular damage. One of the primary effects of high-temperature stress on cells is protein denaturation. In addition, the functions of biological membranes are impaired due to changes in membrane fluidity and properties. These changes result in the generation of reactive oxygen species (ROS) in chloroplasts and mitochondria and the loss of normal cellular functions, which in turn results in cellular damage and consequently cell death (Hoshikawa et al., 2021).

The most important mechanism for thermotolerance at the cellular level is the heat shock response (HSR), which is a process that induces the expression of protective genes in response to heat shock (Ohama et al., 2017). These protective genes consist of functional genes and regulatory genes. Functional genes encode heat shock proteins (HSPs) and detoxification enzymes such as ROS scavengers that function to reduce cellular damage, whereas regulatory genes encode regulatory proteins such as transcription factors. The HSR of plants is regulated by a cascade of transcription factors. In *Arabidopsis*, class A1 heat shock transcription factors (HsfA1s) function as master HSR regulators by inducing the first wave of gene expression in response to heat shock (Liu et al., 2011; Yoshida et al., 2011). HsfA1s activate the expression of not only functional genes but also two important transcription factor genes, *HsfA2* and *DEHYDRATION-RESPONSIVE ELEMENT-BINDING PROTEIN 2A* (*DREB2A*). HsfA2 and DREB2A in turn induce the second wave of gene expression to accomplish substantial cell protection (Sakuma et al., 2006; Charng et al., 2007). Under prolonged heat stress, DREB2A further induces the expression of another transcription factor gene, *HsfA3*, to induce the third wave of HSR (Yoshida et al., 2008). The importance of the heat shock response and these transcription factors in thermotolerance was established by analyses of mutants of these transcription factors (Charng et al., 2007, Sakuma et al., 2006; Yoshida et al., 2008; Yoshida et al., 2011). In tomato, HsfA1a is the master regulator of HSR, and HsfA2 acts as a major Hsf by acting as a coactivator of HsfA1a (Scharf et al., 1998; Mishra et al., 2002). The importance of these Hsfs in the thermotolerance of tomato has also been shown by reverse genetic studies (Fragkostefanakis et al., 2016, Mishra et al., 2002), indicating that HSE-regulated HSR is critical for thermotolerance in tomato. Recently, it was found that heat stress-induced gene transcription in *Arabidopsis* is regulated not only by the Hsf-dependent pathway but also by an Hsf-independent and circadian-regulated pathway, which is in turn regulated by REVEILLE (RVE) 4 and RVE8 (RVE4/8) (Blair et al., 2019; Li et al., 2019). The identification of this new pathway suggests that plants fine-tune their gene expression responses according to different scenarios of heat stress. However, further research is needed to determine how these pathways are used under different heat stress conditions and in different plant species.

Transcriptomic analyses have been used to characterize heat-induced gene expression in tomato (Frank et al., 2009; Fragkostefanakis et al., 2015; Su et al., 2023), and the importance of Hsfs in the expression of chaperones has been demonstrated (Fragkostefanakis et al., 2015). However, how transcriptional pathways are differentially utilized under different heat stress conditions has not been studied in a transcriptome-wide context. Transcriptome analyses have also been employed to identify genes that show differential expression between thermotolerant and thermosensitive tomato accessions in reproductive tissues such as the anthers (Bita et al., 2011), ovules (Bineau et al., 2021) or leaves (Balyan et al., 2020). However, the molecular mechanisms underlying these differences are largely unclear.

To date, various physiological traits have been used to evaluate the thermotolerance of tomato. They include vegetative growth and reproductive traits such as fruit set and yield under field conditions (Bineau et al., 2021; Gonzalo et al., 2021). However, field conditions are not suitable for large-scale screening because of the space and time required to obtain sufficient crop yield and the need to ensure reproducibility over multiple seasons. To overcome this problem, attempts to improve screening efficiency by screening at the seedling or vegetative stages under controlled conditions have been made. In these attempts, physiological traits such as hypocotyl elongation, electrical conductivity, chlorophyll content, survival rates and the Fv/Fm ratio are used (Zhou et al., 2015; Wen et al., 2019; Balyan et al., 2020; Hu et al., 2020). These methods provide data for evaluation that are relatively quantitative, although some of the methods require special equipment and skills for data collection.

Biomarkers are being developed as accurate diagnostic and prediction methods as well as tools to monitor the effects of drugs and therapies in human clinical practice and research. Various types of molecules, such as small molecular compounds, mRNAs, and peptides, are used as biomarkers. One advantage of using biomarkers is that they are physically close to the primary effect or response. For example, a recent report identified expression biomarker genes that reflect the activation of HSF1, which is the major Hsf in humans, to test the effects of drugs on HSF1 activation (Cervantes and Corton, 2021). Since HSR is a fundamental thermotolerance mechanism in tomato, evaluation of HSR functionality is a possible way to assess the acquisition process of basic tolerance at the cellular level. mRNAs are good candidate molecules for use as biomarkers because they are primary products of HSR. However, no expression biomarker genes have been reported to date to evaluate the thermotolerance of tomato cultivars.

In this study, we investigated the relationship between heat tolerance and transcriptional responses in tomato cultivars by evaluating gene expression under various heat stress conditions. We found that both HsfA1-regulated and RVE-regulated pathways were used in response to stepwise heat stress, whereas the HsfA1-regulated pathway played a dominant role in response to acute heat shock. Under acute heat shock stress, a thermotolerant cultivar was able to maintain the HSR, in contrast to thermosensitive cultivars, and we identified candidate expression biomarker genes whose expression levels were correlated with thermotolerance. This study not only indicates the importance of HSR functionality in determining the levels of thermotolerance in tomato seedlings but also provides a new evaluation method for the thermotolerance of different tomato cultivars using expression biomarker genes.

## Materials and methods

### Plant materials and growth conditions

Seeds of tomato (*Solanum lycopersicum* L.) cultivars, ‘Momotaro 8’ (Takii & Co., Ltd., Kyoto, Japan), ‘Arkansas Traveler’ (Reimer Seeds Inc., Maryland, USA), ‘Saturn’ (Takii & Co., Ltd., Kyoto, Japan), and ‘Super First’ (Aisan Seed Co., Ltd., Aichi, Japan) were commercially purchased. Seeds of the cultivar ‘Rouge Grosse Lisse’ were provided by the public repository of plant genetic resources, National Institute of Agrobiological Sciences (NIAS) Gene Bank. Tomato seeds were imbibed in tap water at 25°C for one day under dark conditions. Two or three imbibed seeds were planted in a pot (Plant Pot No. 2, φ70 mm, H60 mm, Yamato Plastic Co., Ltd., Nara, Japan) with a soil mixture of 50% Jiffy-Mix (Sakata Seed Co., Ltd., Yokohama, Japan) and 50% vermiculite (Fukushima Vermi Co., Ltd., Fukushima, Japan). After planting, the pots were placed in a sunlit greenhouse at the University of Tokyo (35°43’N, 139°46’E). Temperatures were set at 25°C from 5:00 AM to 9:00 PM and 20°C from 9:00 PM to 5:00 AM. The relative humidity was set at 60 ± 15% throughout the day. When cotyledons were fully opened, the seedlings were thinned out to ensure one seedling in a pot. Ten days after planting, 1/100 Hoagland solution (Hoagland and Arnon, 1950) was applied to the seedlings.

### Heat stress treatment

Three-week-old seedlings were used for heat stress experiments. Heat stress was applied by transferring the pots into a growth chamber (LPH-240/410S, Nippon Medical & Chemical Instruments Co., Ltd., Osaka, Japan) with relative humidity kept at 60 ± 15% and a photon flux density of 250 ± 25 µmol photons m^-2^ s^-1^.

### Measurement of chlorophyll fluorescence

Before the measurement of chlorophyll fluorescence, seedling pots were placed in the dark for 30 min. Chlorophyll fluorescence images were acquired by using an Open-FluorCam FC 800 (Photon Systems Instruments, Drásov, Czech Republic).

### RNA extraction and qRT−PCR analysis

RNA was extracted from seedling leaves by using the acidic phenol method with RNAiso Plus (Takara Bio, Japan) and the following modifications: Chloroform extraction was conducted twice. To precipitate total RNA, 0.5 volume of a high salt solution (1.2 M NaCl, 0.8 M sodium citrate) was used together with 0.5 volume 2-propanol. After precipitation, RNA solutions were purified with ethanol precipitation. cDNA was synthesized from total RNA by using a High-Capacity cDNA Reverse Transcription Kit (Thermo Fisher Scientific). Transcripts were quantified by using quantitative PCR with a QuantStudio 3 real-time PCR system (Thermo Fisher Scientific) and Power SYBR Green Master Mix (Thermo Fisher Scientific). The amounts of template cDNA were quantified using standard curves, and *18S* rRNA was used as an internal standard. The primers used for the analyses are listed in **Table S1**.

### Microarray analysis

Transcriptomic analysis was performed with a custom oligo microarray (Agilent Technologies), which was designed to detect transcripts of ITAG2.4 gene models. The array design is available at Array Express (A-MTAB-699). Labeling, hybridization and scanning were conducted as described previously (Mizoi et al., 2013). Data were analyzed by using the Subio Platform (Subio Inc., Japan).

### Gene Ontology analysis

GO analysis was conducted with R (ver. 3.2.3) software and the topGO and GO.db packages (Bioconducter version 3.2). For GO annotations of ITAG3.2 proteins, the closest homolog of each protein was identified from *Arabidopsis* TAIR10 proteins by using the Protein–Protein Basic Local Alignment Search Tool (BLASTP) search (National Center for Biotechnology Information BLAST 2.2.29+), and GO annotations of the *Arabidopsis* homolog were assigned to each ITAG3.2 protein. The algorithm and statistical parameters used in topGO were “classic” and “fisher”, respectively.

### Enrichment analysis of upstream sequences

The upstream sequence of the ITAG3.2 genes was retrieved from Phytozome (https://phytozome-next.jgi.doe.gov/) using the BioMart tool on the website, and z values were calculated as previously reported (Maruyama et al., 2012) using the equation


$$\begin{array}{c} z=\frac{N-\mu }{\sigma } \end{array}$$

where N is the observed number in 1-kb upstream sequences of the top 100 upregulated genes, and μ and σ are the mean number and its standard deviation, respectively, in 1-kb upstream sequences of 100 randomly selected genes. Random sampling was repeated 1000 times using all genes available in the microarray.

### Identification of homologs of HsfA1 and RVE4/8 downstream genes

HsfA1 downstream genes were defined as genes that were heat inducible, and their expression was significantly reduced in a quadruple mutant of HsfA1s (Yoshida et al., 2011). The downstream RVE4/8 genes were defined as genes that were heat inducible, and their expression was significantly reduced in a double RVE4/8 mutant (Li et al., 2019). A homolog of each gene was defined as the best hit protein (E-value<1.0E-10) in a BLASTP search against ITAG3.2 proteins for each gene product.

## Results

### Effects of heat stress on physiological characteristics among different tomato cultivars

**Figure 1:**
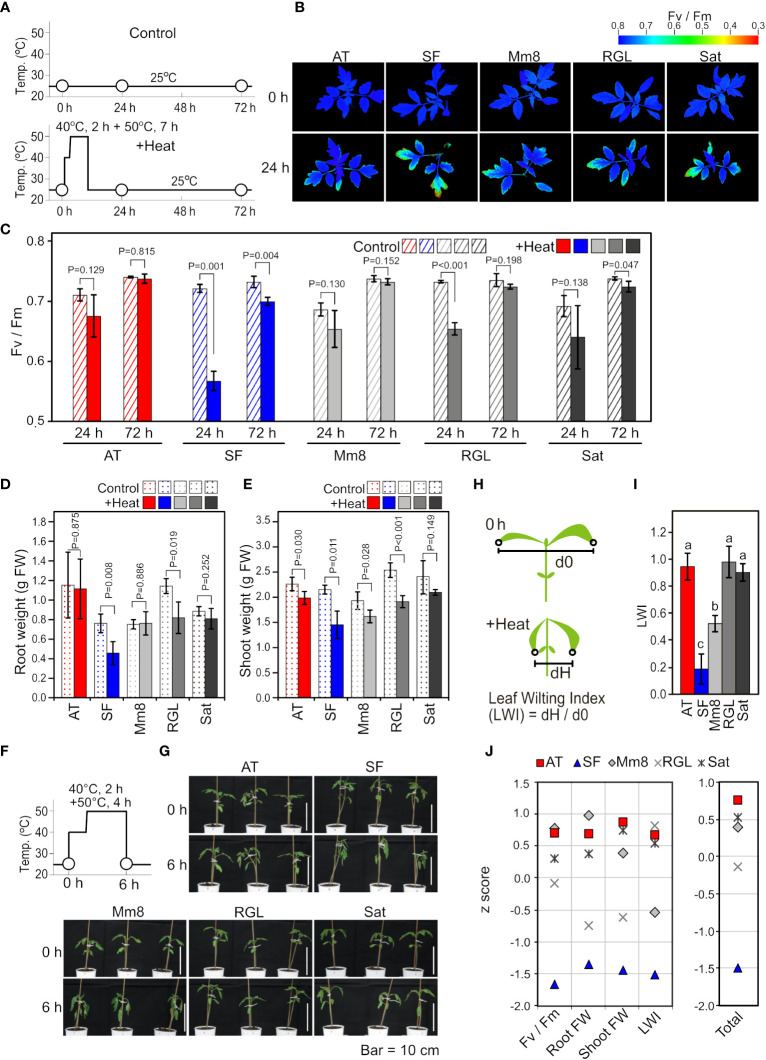
Physiological characteristics among different tomato cultivars. **(A)** Schematic diagram of temperature and duration time for heat stress treatment in **(B-E)**. White circles indicate the time points when Fv/Fm images and values were measured. **(B)** Two-dimensional images of Fv/Fm taken from above the plants before and after heat stress treatment. **(C)** Mean values of Fv/Fm in the two-dimensional images. n=3 to 4. **(D)** Root weights. n=4. **(E)** Shoot weights. n=4. **(F)** Schematic diagram of temperature and duration time for heat stress treatment in **(G)**. White circles indicate the time points when the plants were photographed. **(G)** Horizontal images of plants before and after heat stress treatment. **(H)** Schematic diagram to explain the LWI. **(I)** Mean LWI. n=4. **(J)** z scores for the results of Fv/Fm, root weight, shoot weight and LWI. For Fv/Fm, root weight and shoot weight, the ratios after and before stress were used for calculation. Total, the mean z score of four parameters. In **(C)** to **(E)**, t-tests were used to calculate P values. In **(I)**, values with different letters are significantly different, as determined by Tukey’s HSD tests (p< 0.05).

First, we evaluated the thermotolerance levels of five tomato cultivars based on physiological measurements after heat stress treatment at the seedling stage. Among the five cultivars included in this study, ‘Arkansas Traveler’ (AT) was used as a candidate thermotolerant cultivar (Vavrina et al., 2013). The other four cultivars tested were ‘Super First’ (SF), ‘Momotaro 8’ (Mm8), ‘Rouge Grosse Lisse’ (RGL) and ‘Saturn’ (Sat). As shown in **Figure 1A**, the five cultivars were heat stress treated at 40°C for 2 h and at 50°C for 7 h and then grown at 25°C. The values of the chlorophyll fluorescence parameter Fv/Fm were visualized (**Figure 1B**), and mean values were calculated (**Figure 1C**). The Fv/Fm values tended to decrease compared to the control after 24 h of heat stress in all cultivars, but the reduction was most drastic in SF (**Figures 1B, C**). Regarding growth inhibition after heat exposure, SF and RGL showed decreases in both root and shoot weights when compared to the control (**Figures 1D, E**). We also noticed that extended heat stress caused leaf wilting and that the degree of leaf wilting varied among the cultivars (**Figures 1F, G**). To quantify the magnitude of leaf wilting, we introduced a leaf wilting index (LWI) (**Figure 1H**). The mean value of LWI in SF was lowest among the five cultivars, which indicates that severe leaf wilting was caused by heat stress in SF, whereas the LWIs of AT, RGL and Sat were near 1.0, indicating that leaf wilting was slight in these cultivars (**Figure 1I**). For the evaluation of thermotolerance from multiple perspectives, we integrated the results of physiological data by indexing. First, we normalized the results of various physiological parameters by calculating z scores for each parameter (**Figure 1J**). For all parameters, the z scores of SF were the lowest, while those of AT were among the highest (**Figure 1J**). Finally, to rank the thermotolerance levels of the five cultivars, we averaged these z scores (**Figure 1J**, Total). According to these results, we defined AT and SF as the most thermotolerant and most thermosensitive cultivars, respectively, whereas the other three were defined as cultivars of medium-level thermotolerance.

### Identification of heat-inducible genes and optimization of heat stress conditions to analyze gene expression in response to heat stress

**Figure 2:**
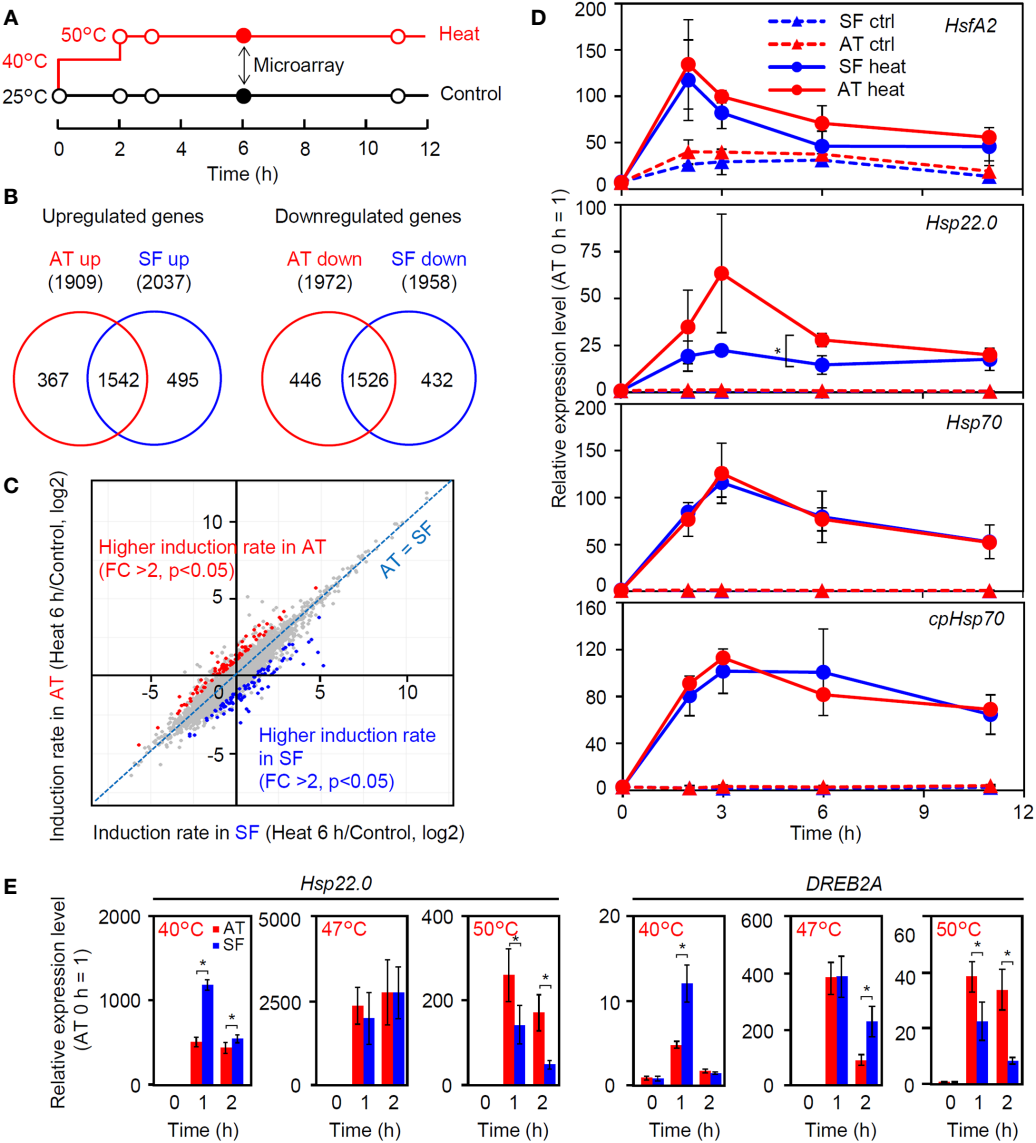
Transcriptomic analysis of thermotolerant and thermosensitive cultivars under the conditions of physiological evaluation. **(A)** Schematic diagram of temperature and duration time for heat stress treatment in microarray and qRT−PCR analyses. Closed circles indicate the sampling point for the microarray analysis. **(B)** Venn diagram for upregulated and downregulated genes in AT and SF. **(C)** Scatterplot showing the heat induction rates of genes in AT and SF. Genes that showed significantly higher induction rates in AT and SF are indicated by red and blue, respectively. **(D)** Time course expression patterns of representative heat-inducible genes in seedlings. Values indicate the mean of three individuals, and the error bars indicate standard deviations. Significant differences between the cultivars are indicated by asterisks (t test, p< 0.05). **(E)** Expression of heat-inducible genes at different stress temperatures. Values indicate the mean of three individuals, and the error bars indicate standard deviations. Significant differences between the cultivars are indicated by asterisks (t test, p< 0.05).

To gain insight into the molecular-level differences among tomato cultivars with different thermotolerances, we compared heat-induced changes in the transcriptomes between AT and SF (**Table S2**). The time point of 6 h of stepwise heat stress (40°C 2 h + 50°C 4 h) was used for the comparison (**Figure 2A**) because a significant difference in leaf wilting was observed between these cultivars at this time point (**Figure 1I**). Approximately 2000 genes were upregulated or downregulated in both cultivars compared to the control (25°C 6 h), and there were large overlaps between the cultivars (**Figure 2B**). Although the Venn diagrams suggested the existence of cultivar-specific genes, the scatter plot showed that most of the genes showed similar responses between the two cultivars under these conditions (**Figure 2C**). There were genes that showed different levels of induction ratio between the cultivars, but the induction ratios of heat-inducible genes were similar between the two cultivars (**Figure 2C**). We next checked the temporal expression patterns of heat stress-related genes that were highly induced in the transcriptomic analysis (**Figure 2D**). The results confirmed that the expression patterns of these genes were similar between the two cultivars over the course of this experiment, except for a stronger expression of *Hsp22.0* at 3 and 6 h in AT than in SF. The temporal patterns observed during the experiment also showed that the expression of these genes peaked before the time point of microarray analysis (6 h), suggesting the necessity of analyzing gene expression in earlier phases of HSR (**Figure 2D**).

### The thermotolerant and thermosensitive cultivars responded differently to weak and severe heat stress

We next analyzed the expression of four heat-inducible genes at 40°C. The expression of these genes peaked at approximately 0.5 to 1 h in both cultivars, and the expression levels at these time points tended to be higher in SF than in AT (**Figure S1**). We next tested the effects of temperature on the early expression responses of representative heat-inducible genes. Similar to the results shown in **Figure S1**, the expression levels of *Hsp22.0* and *DREB2A* were higher in SF than in AT under conditions of 40°C for 1 h (**Figure 2E**). At 47°C, the expression of these genes was similar between the two cultivars (**Figure 2E**). In contrast, at 50°C, the expression levels of these genes were consistently higher in AT than in SF during the 2 h of heat stress, with higher expression at 1 h. It is noted that the expression levels of both genes relative to the 0 h starting point were the highest at 47°C and that the 3°C increase in the temperature from 47°C to 50°C resulted in a marked drop in the peak expression value (**Figure 2E**). This suggests that the heat stress response became stronger under moderate heat stress when the temperature increased from 40°C to 47°C, whereas under severe stress, the temperature increase from 47°C to 50°C resulted in impairment of the ability to maintain the heat stress response. It is possible that the sensitive cultivar SF responded strongly to moderate heat stress, whereas the tolerant cultivar AT retained the ability to maintain HSR under severe heat stress.

### The thermotolerant cultivar retains a high ability to express heat-inducible genes under severe heat stress conditions

**Figure 3:**
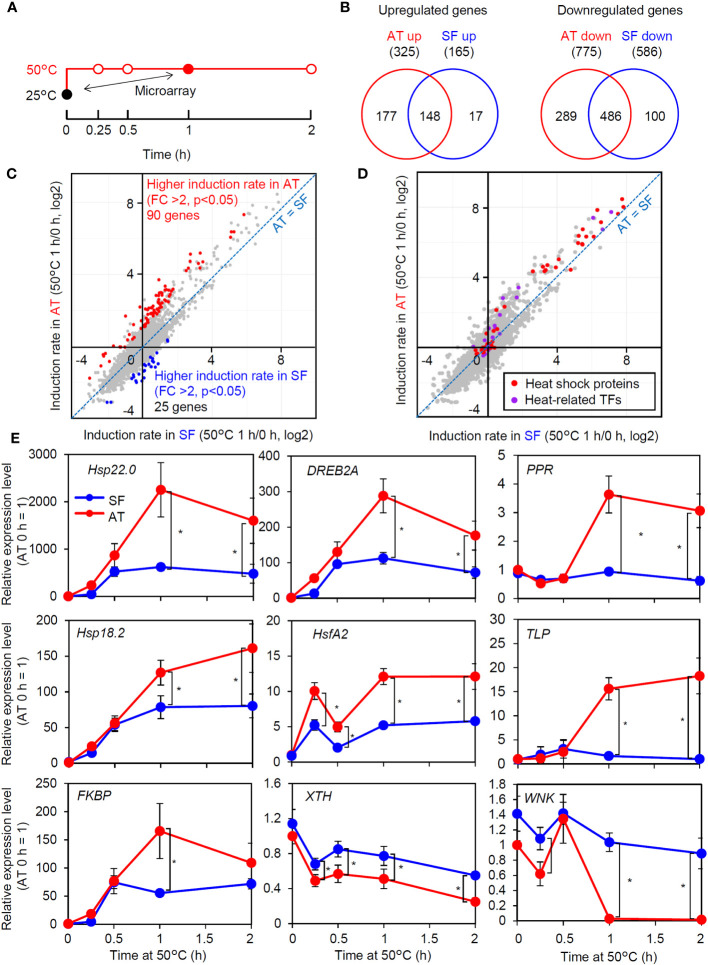
Transcriptomic analysis of thermotolerant and thermosensitive cultivars under acute heat shock. **(A)** Schematic diagram of temperature and duration time for heat stress treatment in the microarray and qRT−PCR analyses. The closed circles indicate the sampling point for the microarray analysis. **(B)** Venn diagram of upregulated and downregulated genes in AT and SF. **(C)** Scatter plot showing the heat induction rate of genes in AT and SF. Genes that showed significantly higher induction rates in AT and SF are indicated by red and blue, respectively. **(D)** Distribution of HSR-related genes in the scatter plot of heat induction rates. **(E)** Time course expression patterns of genes that showed different expression patterns in the transcriptomic analysis. Values indicate the mean of three individuals, and the error bars indicate standard deviations. Significant differences between the cultivars are indicated by asterisks (t test, p< 0.05).

As the response at 50°C was correlated with thermotolerance and was consistently different between the two cultivars (**Figure 2E**), we next tried to confirm whether the observed difference in the strength of gene induction between the two cultivars at 50°C reflected a genome-wide difference. We compared changes in the transcriptome before and after 1 h of severe heat stress at 50°C between AT and SF (**Figure 3A**; **Table S3**). The Venn diagram of upregulated and downregulated genes revealed that larger numbers of genes were induced or repressed in response to heat in AT than in SF. Furthermore, 90% (148/165) of genes that were upregulated in SF were also upregulated in AT, and 83% (486/586) of genes that were downregulated in SF were also downregulated in AT, suggesting a stronger response in AT than in SF (**Figure 3B**). The scatterplot of heat inducibility showed that heat-inducible genes in general were more strongly induced in AT than in SF (**Figure 3C**), and importantly, these strongly induced genes included many HSR-related genes, such as transcription factors (Hsf, DREB2 and MBF2) and HSPs (HSP90, HSP70 and small HSP) (**Figure 3D**). Statistical analysis showed that 90 and 25 genes had twofold higher induction rates in AT and SF, respectively (**Figure 3C**). Gene Ontology (GO) analyses of such differentially expressed genes confirmed that genes related to heat stress were enriched in AT (**Table S4**). In contrast, genes related to lignin metabolism were enriched in SF and were not directly related to the stress response (**Table S5**).

To confirm the results of the transcriptomic analysis, we next analyzed the time course expression profiles of genes that responded differently to heat stress (**Figure 3E**). Heat stress-related genes that had higher induction ratios in AT (*Hsp22.0*, *DREB2A*, *PENTATRICOPEPTIDE REPEAT-CONTAINING PROTEIN (PRR)*, *Hsp18.2*, *HsfA2*, *THAUMATIN-LIKE PROTEIN (TLP)* and *FK506-BINDING PROTEIN* (*FKBP)*) generally showed stronger expression in AT than in SF after 1 h. We also analyzed two genes (xyloglucan endotransglucosylase/hydrolase gene (*XTH*) and WNK family protein kinase gene (*WNK*)) that were repressed by heat and that showed a higher induction rate in SF than in AT. Although the stronger expression of these genes in SF at 1 h was confirmed, these genes tended to be differentially expressed at all time points, including 0 h, suggesting that the detected differences were not directly related to the heat stress response. Collectively, the results of the transcriptome and qRT−PCR analyses suggested that the thermotolerant cultivar AT maintained HSR at 50°C better than the thermosensitive cultivar SF.

### Differential regulation of heat-inducible gene expression between stepwise heat stress from 40°C to 50°C over 6 h and acute heat shock of 50°C for 1 h

**Figure 4:**
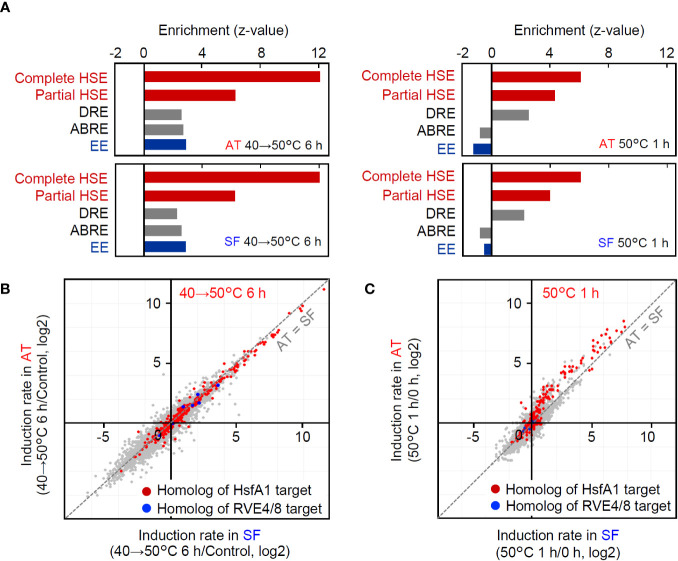
Comparison of gene regulation between the two conditions of the transcriptomic analyses. **(A)** Enrichment of cis-acting elements in the 1-kb upstream sequence of the top 100 upregulated genes under each condition and cultivar. Enrichment of each element is shown as the z value against the expected number and SD in the 1-kb upstream sequence of 100 randomly selected genes. Complete HSE, (nGAAn)(nTTCn)(nGGAn); Partial HSE, (nGAAn)(nTTCn); DRE, A/GCCGAC; ABRE, ACGTGG/T; EE, AAAATATCT. **(B, C)** Response of HsfA1 target gene homologs and RVE4/8 target gene homologs at 40°C to 50°C for 6 h **(B)** and 50°C for 1 h **(C)**, respectively. HsfA1 target genes and RVE 4/8 target genes were defined according to Yoshida et al. (2011) and Li et al. (2019). Homologs were defined as the best hits in BLASTP searches of gene products.

In the transcriptomic analyses under the two conditions, AT and SF similarly responded to stepwise heat stress from 40°C to 50°C over 6 h, whereas the induction of heat-inducible genes was weaker in SF than in AT under acute heat shock at 50°C for 1 h. To further investigate the differences in gene expression profiles between the two heat stress conditions, we analyzed the microarray data under the two conditions. First, we conducted an enrichment analysis of cis-acting elements in the promoters of the top 100 upregulated genes under each tested set of conditions and in each cultivar. Among the transcription factors that regulate heat-responsive gene expression, Hsfs recognize the heat shock element (HSE) as a cis-acting element, whereas RVE4/8 recognize the evening element (EE). The dehydration-responsive element (DRE) is the target of DREB2A, which acts downstream of Hsfs (Yoshida et al.). The abscisic-acid responsive element (ABRE), which is related to the abscisic acid response, was also analyzed as another major stress-responsive cis-acting element. The results revealed that HSE and DRE were significantly enriched under both conditions (**Figure 4A**). In contrast, EE and the abscisic acid-responsive element (ABRE) were enriched only under the stepwise heat stress condition of 40°C to 50°C for 6 h (**Figure 4A**). This tendency was similar between the cultivars. These results suggest that HSE and DRE are commonly used under both stepwise and acute heat stress conditions, whereas EE and ABRE are used only under stepwise conditions. The different tendencies of cis-acting element enrichment suggest that heat-responsive signaling pathways are used differently under these conditions.

Then, we also checked the expression of homologs of HsfA1 target genes (Yoshida et al., 2011) and RVE4/8 target genes. Consistent with the results of the enrichment analysis (**Figure 4A**), both HsfA1 target homologs and RVE4/8 target homologs were included in heat-responsive genes at 40°C to 50°C for 6 h (**Figure 4B**). In contrast, HsfA1 target homologs, including *DREB2A*, were induced at 50°C for 1 h, but RVE4/8 target homologs were not induced under these conditions (**Figure 4C**). Furthermore, the induction rates of HsfA1 target homologs were generally higher in AT than in SF (**Figure 4C**). These results suggest that the HsfA1-HSE and its downstream DREB2-DRE pathways are commonly used under both stepwise and acute stress conditions, whereas the RVE4/8-EE pathway is used under stepwise heat stress conditions but does not function under acute heat stress conditions. Furthermore, it was confirmed that the differential gene expression between AT and SF at 50°C for 1 h was due to the difference in the functionality of the HsfA1-regulated HSR pathway.

### Selection of candidates for expression biomarker genes that correlate with thermotolerance

**Figure 5:**
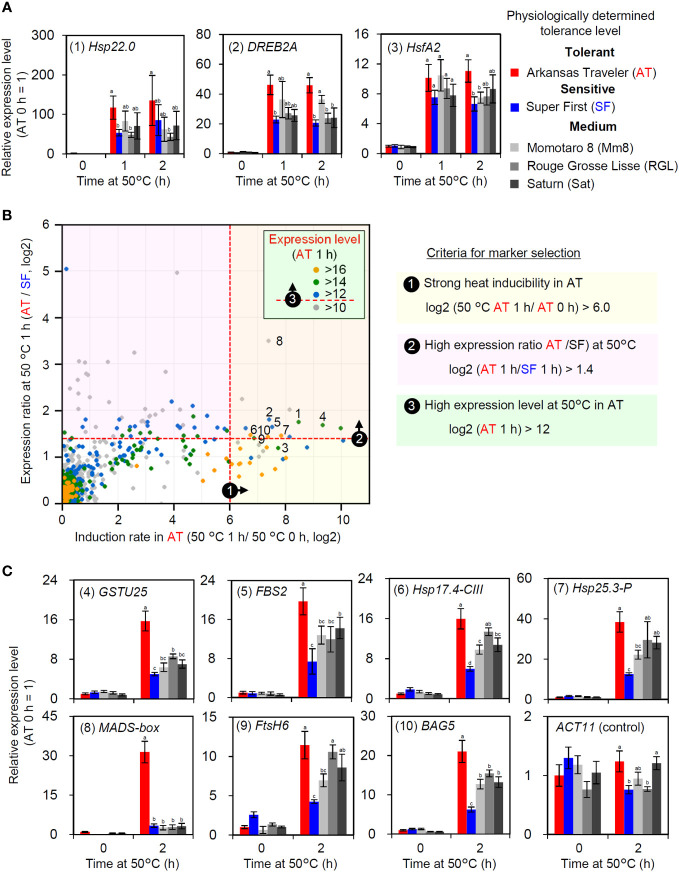
Selection and evaluation of expression biomarker genes. **(A)** Expression levels of HSR-related genes in five cultivars with different thermotolerance levels. Numbers before gene names are identifiers for expression biomarkers. **(B)** Criteria and results of expression biomarker selection. Candidates of expression biomarkers were selected based on three criteria. Validated expression biomarker genes are indicated by identifier numbers from 1 to 10. **(C)** Validation of selected expression biomarkers using the five cultivars by qRT−PCR. In **(A, C)**, the values indicate the mean of three individuals, the error bars indicate standard deviations, and values with different letters are significantly different (Tukey’s HSD test, p<0.05).

As the expression levels of HSR-related genes were correlated with thermotolerance in AT and SF, we next aimed to confirm whether such a correlation applied to other cultivars. Among the five tomato cultivars used in this study, AT and SF were the most thermotolerant and the most thermosensitive cultivars, respectively, whereas the other three had medium levels of thermotolerance (**Figure 1**). We analyzed the expression of representative HSR-related genes at 50°C using the five cultivars and confirmed that the three medium-level thermotolerant cultivars showed intermediate levels of responses between AT and SF (**Figure 5A**). This suggests that the genes that were more strongly induced in AT than in SF could be used as expression biomarker genes for thermotolerance. As primary candidates for the expression biomarker, we selected these three genes and numbered them from 1 to 3 (**Figure 5A**; **Table S6**).

Then, we selected additional candidates for expression biomarker genes from the results of transcriptomic analysis at 50°C. We used three criteria for the selection (**Figure 5B**). First, since strong inducibility in response to heat is important for optimal sensitivity, genes that showed high induction rates in AT were selected (log_2_ (AT 1 h/AT 0 h) > 6). The second criterion was that the expression levels at 50°C should be higher in AT than in SF (log_2_ (AT 1 h/SF 1 h) > 1.4). Third, because high absolute expression levels increase the reliability of RT−PCR results, genes that showed high expression levels at 50°C were selected (log_2_ (AT 1 h) > 12). According to these criteria, seven additional candidate genes were selected (numbered 4 to 10; **Figure 5B**; **Table S6**). The expression of these genes was analyzed to confirm their utility. As more stable responses were observed at the 2 h point than at the 1 h point during the heat tolerance experiment (**Figure S2**), we analyzed the expression responses of these genes at 2 h. AT and SF had the highest and lowest expression levels of most genes, respectively, whereas the other three cultivars showed intermediate levels of expression (**Figure 5C**). A MADS-box gene showed exceptionally strong expression in AT (**Figure 5C**, (8)). Through gene selection and validation, ten genes were identified as potential expression biomarker genes whose expression levels correlate with thermotolerance (**Table S6**).

### Development of a scoring method for estimating the thermotolerance of tomato cultivars using expression biomarker genes

**Figure 6:**
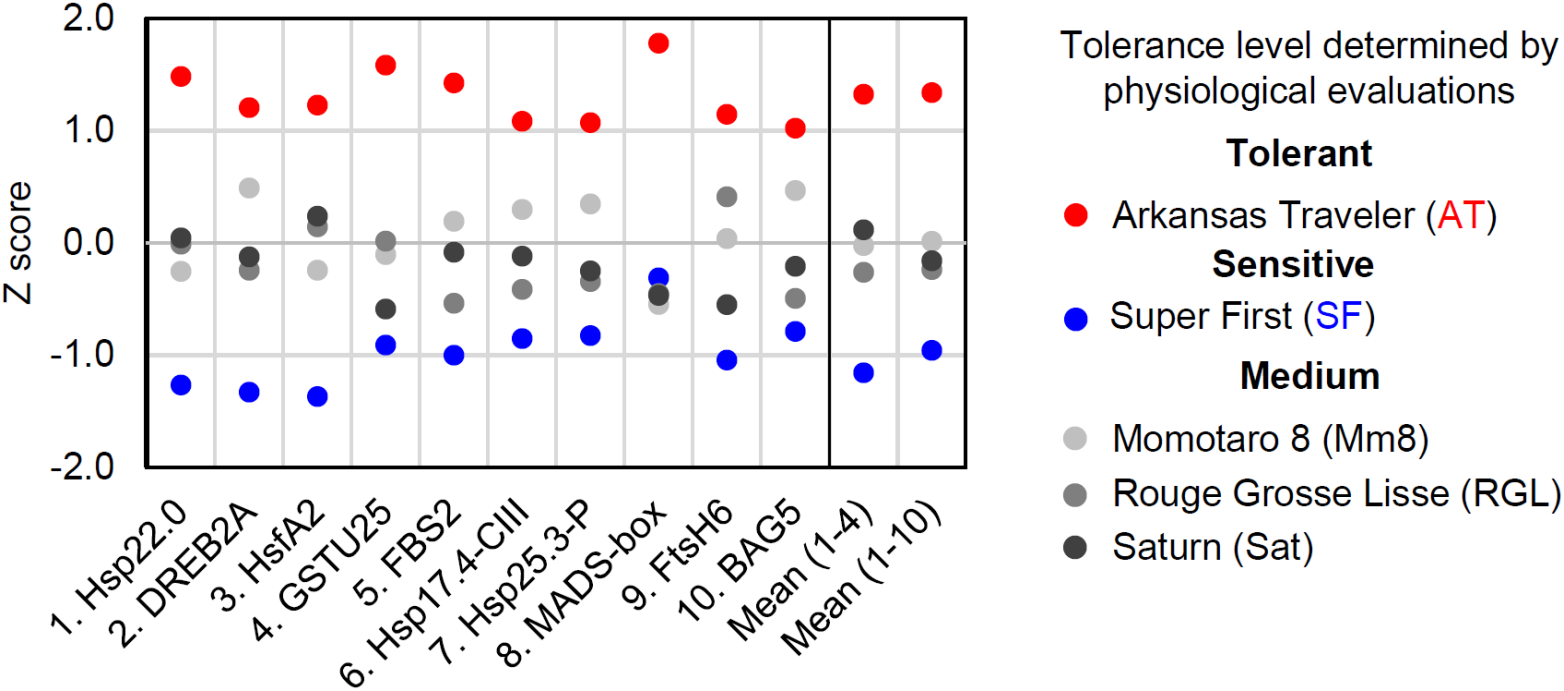
Scoring of biomarker expression among different cultivars. The expression levels of the ten biomarker genes at 50°C for 1 h were normalized, and their z scores were calculated for each gene. The mean z scores of four or ten biomarker genes were calculated for each cultivar.

We next developed a scoring method for the estimation of tomato cultivar thermotolerance. The results of gene expression analysis vary from gene to gene, and qRT−PCR data include deviations and errors. One method to overcome these problems is to measure the expression of multiple biomarker genes and to average the results. We first standardized the qRT−PCR results from the 2 h time point and calculated a z score for each cultivar (**Figure 6**). Then, mean z scores for each cultivar were calculated from the results of four (gene 1 to 4) or ten (gene 1 to 10) genes (**Figure 6**). These calculations revealed that AT and SF had the highest and lowest mean z scores, respectively, and that the other three cultivars had medium z scores. This result was consistent with the results of the physiological evaluation (**Figure 1**), showing the usefulness of this scoring method in estimating the thermotolerance of tomato cultivars.

## Discussion

**Figure 7:**
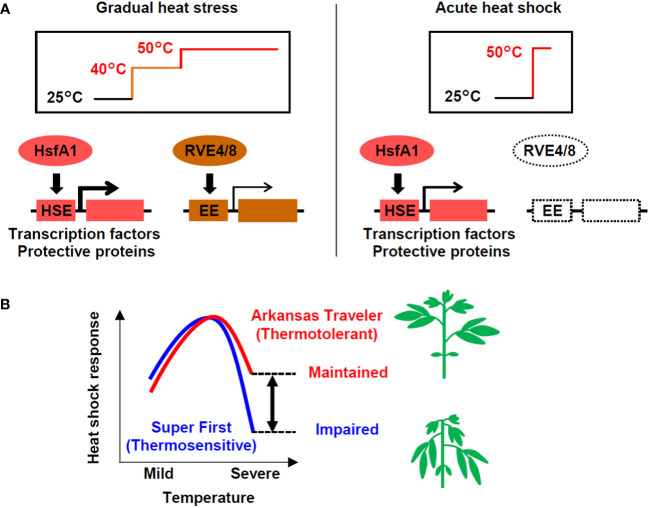
Models of the heat shock response in tomato at different temperatures in different cultivars. **(A)** Under gradual heat stress, both the HsfA1-HSE and RVE4/8-EE regulated pathways are activated. In contrast, in response to acute heat shock, the HsfA1-HSE-regulated pathway, which activates the expression of heat-related transcription factors (e.g., HsfA2, DREB2A, MBF1c) and protective proteins (e.g., HSPs, GST), plays a dominant role. **(B)** The heat shock response regulated by the HsfA1-HSE pathway is impaired under severe high temperatures, but thermotolerant cultivars have a greater ability to maintain the response. The differences in the response at severe temperatures (double-headed arrow), which can be measured using expression marker genes, can potentially be used to evaluate thermotolerance. The heat shock response levels represent the conceptualization of the data shown in **Figures 2E**, **5A, C**.

With the aim of examining the possibility of evaluating the thermotolerance of tomato based on gene expression, we compared gene expression in response to heat stress among cultivars with different levels of thermotolerance. We first found differential usage of transcriptional pathways under different heat stress regimes: both the HsfA1- and RVE-regulated pathways are utilized under stepwise heat stress conditions, while the HsfA1-regulated pathway plays a dominant role under severe heat shock stress, such as that imposed by a temperature of 50°C (**Figure 7A**). We also found that thermotolerant cultivars could maintain the induction of heat-inducible genes even when directly treated with severe heat stress (**Figure 7B**). According to these results, we developed expression biomarker genes whose expression levels under severe heat stress conditions were correlated with the thermotolerance levels of the cultivars (**Figure 7B**). This study reveals how transcriptional pathways are differentially regulated in response to different heat stress scenarios and among cultivars that differ in thermotolerance.

The expression of heat stress-inducible genes is a key response to heat shock, which is necessary for cellular survival under high temperatures. HsfA1s function as master regulators of this pathway via the cis-acting element HSE (Liu et al., 2011; Yoshida et al., 2011). In addition to the HsfA1-dependent pathway, recent research has revealed the existence of an HsfA1-independent pathway that is regulated by the circadian regulators RVE4/8 via the cis-acting element EE (Li et al., 2019). This pathway induces specific genes, such as the transcription factors ERF53 and ERF54. In our study, the comparison of transcriptome data for conditions of stepwise heat stress (from 40°C to 50°C over 6 h) and conditions of acute heat shock (50°C 1 h) suggested that the HsfA1-HSE pathway is used under both scenarios, while the RVE4/8-EE pathway was only active under the stepwise heat stress conditions (**Figure 4**). Because both experiments were conducted during the daytime, when the RVE4/8-EE pathway can function (Li et al., 2019), this result can be interpreted as indicating that tomato plants respond differently to heat stress depending on the temperature conditions (**Figure 7A**). In *Arabidopsis*, the RVE4/8-EE pathway is rapidly activated under nonlethal heat stress conditions and is thought to play a role in integrating temperature and circadian responses to prepare plants for exposure to high temperatures during the day (Li et al., 2019). The stepwise stress conditions examined in this study were nonlethal, although the time scale was long. It is reasonable to assume that under these conditions, the tomato plants prepared themselves for more severe daytime heat via the RVE4/8-EE pathway.

In contrast, it is possible that the application of acute stress at 50°C for 1 h in our study created conditions that could cause the exclusive induction of the HsfA1-dependent heat shock response without the influence of the circadian-regulated pathway. Acute stress at 50°C without acclimation is lethal and may require urgent cell protection for survival. Although such heat stress is unlikely to occur in natural environments, it serves as a model of lethal heat stress. Under these conditions, the thermotolerant cultivar AT and the thermosensitive cultivar SF showed differential gene expression. The genes that were more strongly induced in AT included many HSR-related genes, such as those for key transcription factors and HSPs (**Figure 3D**). Consistently, homologs of HsfA1 target genes generally showed stronger induction in AT (**Figure 4D**). These results show that under acute heat stress conditions, the thermotolerant cultivar AT was able to induce stronger HSR via the HsfA1-HSE pathway. The peak expression levels of representative regulatory and functional genes (i.e., *DREB2A* and *Hsp22.0*) relative to those at the 0 h starting time were decreased more markedly at 50°C than at 47°C, indicating partial impairment of the HSR (**Figures 2E**, **7B**). This suggests that the difference in thermotolerance between the AT and SF cultivars is related to the ability to retain functionality of the HsfA1-HSE pathway to protect cells from heat shock even under severe heat stress conditions, such as a temperature of 50°C (**Figure 7B**).

In the gene expression analysis using the five cultivars, the expression levels at 50°C of the two transcription factor genes *HsfA2* and *DREB2A*, which are induced in response to heat stress through the action of the master regulator HsfA1 (Scharf et al., 1998; Sakuma et al., 2006; Charng et al., 2007; Ohama et al., 2017), correlated well with thermotolerance (**Figure 5A**). The other genes whose expression at 50°C was correlated with thermotolerance include functional genes that encode protective proteins such as small HSPs (sHSPs), glutathione S-transferase (GST), an FtsH protease and a Bcl-2-associated athanogene (BAG) family protein (**Figures 5A, C**) (Żelisko et al., 2005; Labrou et al., 2015; Sedaghatmehr et al., 2016; Mano et al., 2019; Irfan et al., 2021). Considering the importance of these regulatory and functional genes in the HSR that are regulated by HsfA1, it is unsurprising that the expression levels of these genes under severe heat stress conditions correlate well with the levels of thermotolerance.

Transcriptome analyses have been used to identify genes that are differentially expressed between thermotolerant and thermosensitive tomato cultivars. Balyan et al. (2020) found many genes that were differentially regulated the leaves of thermotolerant versus thermosensitive Indian cultivars, and several of them were shown to be involved in thermotolerance by reverse-genetic experiments. However, the differentially regulated genes identified here include accession-specific genes that may not be related to heat stress. In this study, we pursued the identification of general factors that can be universally utilized regardless of genetic origin; to this end, we optimized stress conditions to cause global transcriptional changes and finally validated gene expression patterns using five accessions. In addition, we demonstrated the involvement of the HsfA1-HSE pathway in the observed differences. Other relevant studies include those conducted using reproductive tissues under mild stress conditions. In the ovules of tomato cultivated under high-temperature field conditions, some identified candidate genes in QTLs associated with yield stability under heat stress showed differential expression between thermotolerant and thermosensitive lines, and these genes were enriched in HSPs (Bineau et al., 2021). In another study, thermotolerant tomato cultivars tended to express HSPs more strongly in the anthers than sensitive cultivars did under normal conditions, and their expression was stronger in the thermosensitive cultivars under prolonged intermediate heat stress conditions at 32°C (Bita et al., 2011). These results differ from those of the present study in the tissue examined and the strength and duration of heat stress. However, considering the results of the present study along with these other studies, it is suggested that the HSR has general importance in determining thermotolerance, although its usage can be altered depending on tissues, growth stages and heat stress conditions.

In the physiological evaluation of thermotolerance, the thermosensitive cultivar SF showed severe wilting of leaves and a severe reduction in Fv/Fm after prolonged exposure to conditions of 50°C (**Figures 1B, C, G, I**). The wilting of leaves indicates severe water loss in SF. The observation that the reduction in Fv/Fm tended to appear in the tips rather than in the basal parts of the leaves is consistent with the idea that water loss exacerbates cellular damage. Water loss might exacerbate cellular damage either by loss of transpiration-mediated cooling or organellar malfunction induced by dehydration. The relationship between water loss and cellular damage suggests that the ability to maintain leaf water content is important for thermotolerance. The causal relationship between the loss of the ability to retain HSR and the loss of the ability to maintain cellular water content is not clear at this time; i.e., insufficient HSR might impair leaf cell integrity and cause water loss, but conversely, limited water supply, possibly due to root damage or a low root water uptake capacity, might cause leaf cell dehydration and impaired cellular activities, including those related to HSR. Mechanisms to determine these differences should be elucidated in the future to improve the thermotolerance of cultivated plants.

Here, we identified expression biomarkers whose expression correlates with thermotolerance in tomato (**Figure 5**; **Table S6**). One example of how expression biomarker genes that were identified in this study or that will be developed in other crops may be used is in the selection of thermotolerant cultivars or lines. Gene expression analyses enable evaluation of the cellular stress response independent of morphological variations. Another example is the optimization of growth conditions in high-temperature environments. The strength of stress under different conditions in a single cultivar can also be evaluated by expression analysis of the identified biomarker genes. In modern facility horticulture, stress strength can be controlled by changing air or soil temperature, water supply or soil composition, but control of conditions such as cooling requires much energy. By finding a limit of high temperature that does not cause attenuation of the HSR, it is possible to optimize costs for facility horticulture in high temperature-prone environments. The expression biomarker identified here can also be used for the identification of expression QTLs (eQTLs) that are related to HSR. Determination of such eQTL genes will contribute to a basic understanding of HSR or thermotolerance mechanisms.

An advantage of using expression biomarker genes over physiological parameters is its generality. Morphological variability among cultivars, such as the initial size of plants and proportion of organs, makes the accurate evaluation of physiological traits such as growth parameters difficult. In contrast, the measurement of expression marker genes can enable monitoring of cellular responses and is less affected by morphological differences, which suggests the usefulness of using expression biomarker genes to evaluate morphologically divergent cultivars. Furthermore, the measurement of the expression of biomarker genes can be performed using standard laboratory equipment and methods for molecular biological analysis using a qRT−PCR apparatus (**Figures 5A, C**). Although we found that Fv/Fm values were correlated with thermotolerance indicated by other parameters (**Figures 1B, C, J**) and that although these values have proven to be useful indicators of early heat stress (Zhou et al., 2015), an imaging chlorophyll fluorometer, which is not a widely used piece of equipment, was necessary for accurate analysis due to the nonuniform decrease in Fv/Fm in a leaf (**Figures 1B, C**).

In summary, we analyzed heat stress-inducible gene expression in thermotolerant and thermosensitive tomato cultivars and developed a method to identify expression biomarker genes that are associated with thermotolerance using tomato seedlings as a model. We noted that the HsfA1-regulated HSR played a dominant role in the response to severe heat shock and that the ability to activate HSR under such conditions was associated with the thermotolerance of cultivars (**Figure 7**). The gene expression scores of candidate biomarker genes that were selected based on transcript data were correlated with thermotolerance, underscoring the usefulness of biomarker genes in the evaluation of thermotolerance. Since the HsfA1-HSE pathway is conserved among plants, it is expected that the findings obtained in this study can be used to understand the heat stress response and to evaluate tolerance in tomato as well as other crops.

## Accession numbers

ACT11 (Solyc10g080500), BAG5 (Solyc10g084170), cpHSP70 (Solyc11g020040), DREB2A (Solyc05g052410), FBS2 (Solyc02g079150), FKBP (Solyc09g092690), FtsH6 (Solyc02g081550), GSTU25 (Solyc07g056510), HsfA2 (Solyc08g062960), Hsp17.4-CIII (Solyc03g123540), Hsp18.2 (Solyc09g015000), Hsp22.0 (Solyc03g113930), Hsp25.3-P (Solyc03g082420), Hsp70 (Solyc03g117630), MADS-box (Solyc01g098070), PPR (Solyc05g051340), TLP (Solyc02g083790), WNK (Solyc08g082980), XTH (Solyc11g066270)

## Data availability statement

The datasets presented in this study can be found in online repositories. The names of the repository/repositories and accession number(s) can be found in the article/**Supplementary Material**.

## Author contributions

JM: Conceptualization, Data curation, Funding acquisition, Investigation, Methodology, Supervision, Writing – original draft. DT: Conceptualization, Data curation, Investigation, Methodology, Writing – original draft. TI: Investigation, Methodology, Writing – review & editing. SK: Investigation, Writing – review & editing. TS: Investigation, Writing – review & editing. KK: Investigation, Methodology, Writing – review & editing. HT: Conceptualization, Investigation, Writing – review & editing. KS: Supervision, Writing – review & editing. KY-S: Conceptualization, Data curation, Funding acquisition, Project administration, Supervision, Writing – review & editing.
