# Supplementary material for: The ability to induce heat shock transcription factor-regulated genes in response to lethal heat stress is associated with thermotolerance in tomato cultivars
Source: Front Plant Sci. 2023 Oct 5;14:1269964. doi: 10.3389/fpls.2023.1269964 (PMC10585066; doi:10.3389/fpls.2023.1269964)
Supplement: Supplementary file 1 [file DataSheet_1.pdf]

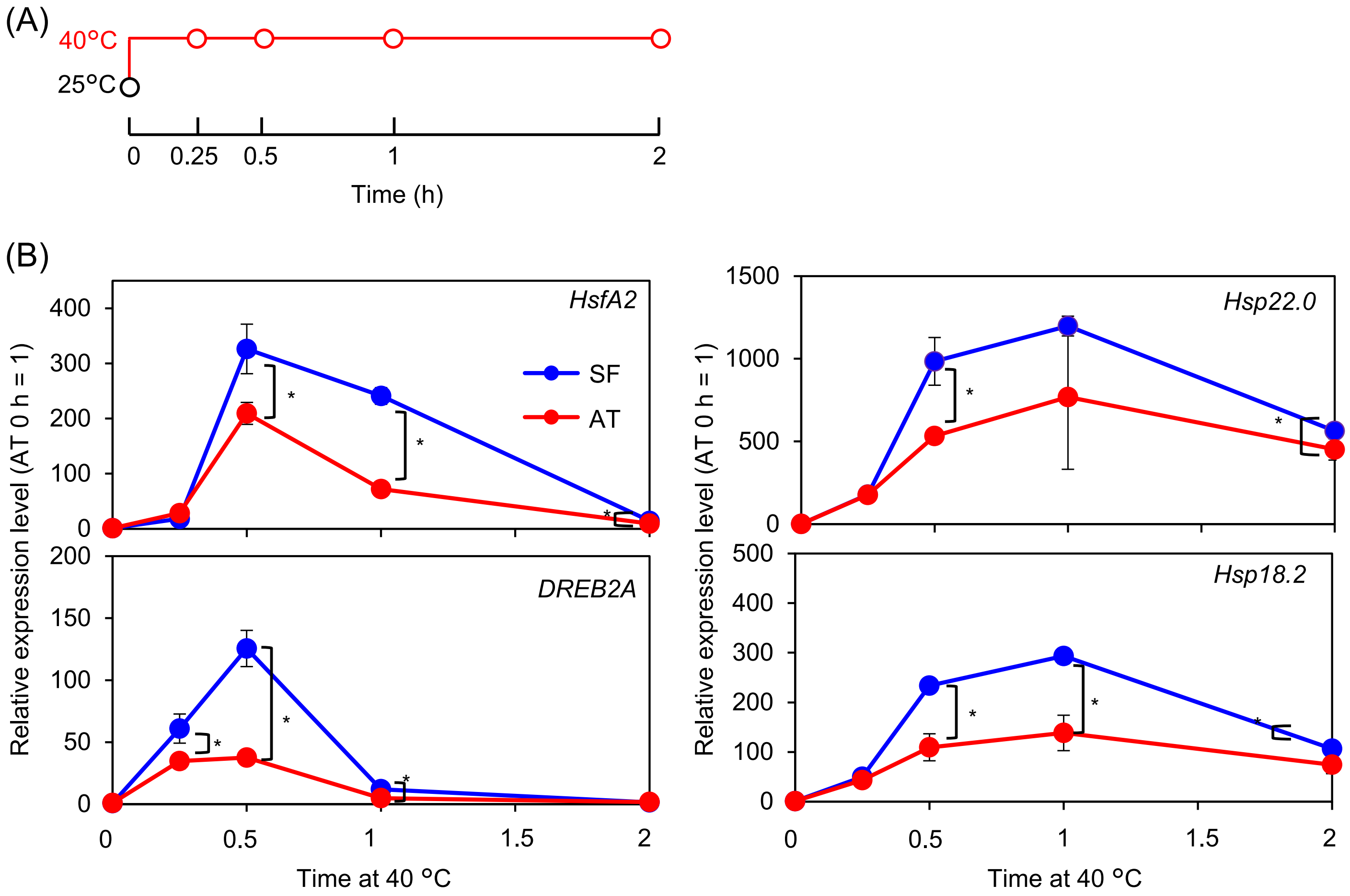

Figure S1. Expression of heat-inducible genes at 40°C over the course of the experiment.
Expression time course of representative heat-inducible genes during 2 h of relatively mild heat stress in AT and SF was analyzed by qRT-PCR. (a) Time duration. (b) Quantification results. Values indicate means of triplicate measurements, and the error bars indicate standard deviations. Significant differences between the cultivars are indicated by asterisks (t-test, $p < 0.05$).

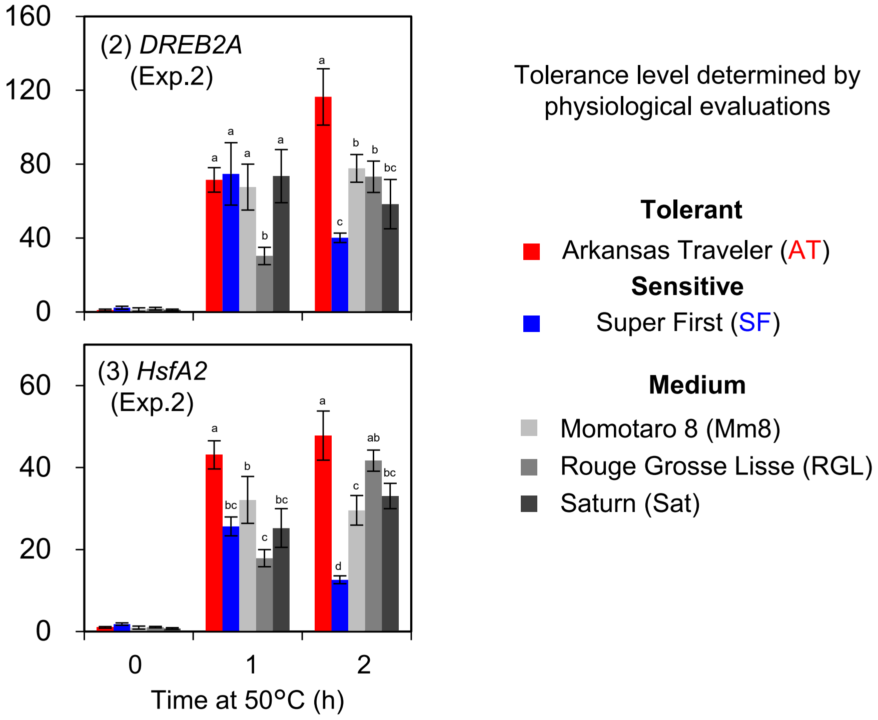

Figure S2. Repeated of the experiment shown in Figure 5A.
Values indicate means of triplicate measurements, and the error bars indicate standard deviations. Significant differences between the cultivars are indicated by different characters (t-test, $p < 0.05$).

Table S1. Primers used in this study

ITAG2.4 locus	Gene name	qRT-PCR primers	Marker ID
Solyc10g080500	*ACT11*<br>(ACTIN11)	TCAGCCACACAGTTCCCATC<br>TGAGATCACGCCCTGCAAG	Control
Solyc03g113930	*Hsp22.0*<br>(Small heat shock protein)	TTTCCTCCCGAAGATCCTTT<br>TTCCTTCTTCATCCCTGGAA	1
Solyc05g052410	*SlDREB2A*<br>(Dehydration-responsive element-binding protein 2A)	CTGCGGCAAGACATGAAGATAG<br>CTGACACCTCCTTGGTCTCGTC	2
Solyc08g062960	*SlHsfA2*<br>(Heat shock transcription factor A2)	CGAGCAGCAGAGGAAGTAGTG<br>TCACCCCATTCAGGTGTTTTC	3
Solyc07g056510	*GSTU25*<br>(Glutathione S-transferase U25)	AGCAACAAGAGCTCATTGCTCCTA<br>CAATGTACTGAACAATGATGAGGG	4
Solyc02g079150	*FBS2*<br>(F-BOX STRESS INDUCED)	CCAGAAAGACGGTTGGTTTC<br>CCAGAAAGACGGTTGGTTTC	5
Solyc03g123540	*Hsp17.4-CIII*<br>(Small heat shock protein)	ACGCTGGTGATACGAAGCAA<br>CATTGCAGTAATCGGGCAGC	6
Solyc03g082420	*Hsp25.3-P*<br>(Small heat shock protein)	AGCGTTGGTGGAACCTCTAAG<br>CTTTGTTATCGCCGGTAGCC	7
Solyc01g098070	*MADS-box*<br>(MADS-box transcription factor)	TTCCATCTGTATCCGCCCAG<br>TTGACCCCAAAGATTCACCAG	8
Solyc02g081550	*FtsH6*<br>(Plastid metalloprotease)	TCTGTCTCTCAGTTCCCCC<br>GTACTCAACAGCTTCCTCCTG	9
Solyc10g084170	*BAG5*<br>(Bcl-2-associated athanogene)	ACCAGTACAATCGGTCCACG<br>CTGGATCGGAACTGACGGAG	10
Solyc03g117630	*Hsp70*<br>(Heat shock protein 70)	GGGAAATTGGATCCATCTGA<br>CCAGCTCCACCTTGGTACAT	
Solyc11g020040	*CpHsp70*<br>(Chloroplast heat shock protein 70)	GAGCTCAAGGATGCCATTTC<br>CAGATGATCCAGTTGTACCAG	
Solyc05g051340	*PPR*<br>(Pentatricopeptide repeat- containing protein)	TTGATGGACGAAATGGAGTG<br>GAAGGAGCCCATTTCCTATC	
Solyc09g015000	*Hsp18.2*<br>(Small heat shock protein)	CAAGTGTTTGATGAAATTTCC<br>ATGCTAGTAGAAGAAGACCAC	
Solyc02g083790	*TLP*<br>(Thaumatin-like family protein)	CTCCTACTGGATTTCAGCTG<br>AAGTGAATTTACCCGTTGCC	
Solyc09g092690	*FKBP*<br>*(FK506-binding proteins)*	GAAGGAAGCCATGGATGAGG<br>CATCCCCATCCCCATATCC	
Solyc11g066270	*XTH*<br>(Homolog of AtXTH32)	AGCGCCTTCTAGCTCTCCTTC<br>AGTTCCTATGCACCCACAAC	
Solyc08g082980	*WNK*<br>(WNK family protein kinase)	CACAAAGCTTGCCAAAGTCG<br>GTTTCTCCCATTTCGTCTCAC	
	18S rRNA	AGACGAACAACTGCGAAAGC<br>AGCCTTGCGACCATACTCC	

Table S4. Gene Ontology analysis of 251 genes that showed a higher induction rate in AT than in SF ($\log_2$(AT 1 h/AT 0 h) – $\log_2$(SF 1 h/SF 0 h) >1.5).

Rank	GO.ID	Term	Annotated	Significant	Expected	P value
1	GO:0009408	response to heat	233	19	1.68	6.30E-15
2	GO:0006950	response to stress	3647	59	26.32	2.30E-10
3	GO:0009266	response to temperature stimulus	636	22	4.59	1.10E-09
4	GO:0006457	protein folding	282	13	2.04	1.40E-07
5	GO:0050896	response to stimulus	6511	76	46.99	6.00E-07
6	GO:0009628	response to abiotic stimulus	2299	37	16.59	2.00E-06
7	GO:0009644	response to high light intensity	70	6	0.51	1.10E-05
8	GO:0006952	defense response	1544	26	11.14	4.20E-05
9	GO:0009617	response to bacterium	547	14	3.95	4.40E-05
10	GO:0009615	response to virus	111	6	0.8	0.00016
11	GO:0009607	response to biotic stimulus	1211	21	8.74	0.00017
12	GO:0010187	negative regulation of seed germination	41	4	0.3	0.00021
13	GO:0010035	response to inorganic substance	1061	19	7.66	0.00023
14	GO:0042742	defense response to bacterium	420	11	3.03	0.00024
15	GO:0098542	defense response to other organism	808	16	5.83	0.00025
16	GO:0043207	response to external biotic stimulus	1178	20	8.5	0.00032
17	GO:0051707	response to other organism	1178	20	8.5	0.00032
18	GO:0050832	defense response to fungus	298	9	2.15	0.00032
19	GO:0009642	response to light intensity	135	6	0.97	0.00045
20	GO:0009605	response to external stimulus	1527	23	11.02	0.00061

Table S5. Gene Ontology analysis of 209 genes that showed a higher induction rate in SF than in AT ($\log_2$(SF 1 h/SF 0 h) – $\log_2$(AT 1 h/AT 0 h) > 1.5).

Rank	GO.ID	Term	Annotated	Significant	Expected	P value
1	GO:0046271	phenylpropanoid catabolic process	21	3	0.08	6.20E-05
2	GO:0046274	lignin catabolic process	21	3	0.08	6.20E-05
3	GO:0009809	lignin biosynthetic process	128	5	0.47	0.00011
4	GO:0055114	oxidation-reduction process	1570	16	5.82	0.00017
5	GO:0009808	lignin metabolic process	149	5	0.55	0.00023
6	GO:1902600	hydrogen ion transmembrane transport	113	4	0.42	0.00083
7	GO:0009699	phenylpropanoid biosynthetic process	202	5	0.75	0.00093
